# Anatomical evaluation of CT-MRI combined femoral model

**DOI:** 10.1186/1475-925X-7-6

**Published:** 2008-01-30

**Authors:** Yeon S Lee, Jong K Seon, Vladimir I Shin, Gyu-Ha Kim, Moongu Jeon

**Affiliations:** 1 School of Information and Mechatronics, Gwangju Institute of Science and Technology, Gwangju, Korea; 2 Center for Joint Disease, Chonnam National University Hwasun Hospital, Hwasun-gun, Jeonnam, Korea; 3 Division of Automotive & Mechanical Engineering, Nambu University, Gwangju, Korea

## Abstract

**Background:**

Both CT and MRI are complementary to each other in that CT can produce a distinct contour of bones, and MRI can show the shape of both ligaments and bones. It will be ideal to build a CT-MRI combined model to take advantage of complementary information of each modality. This study evaluated the accuracy of the combined femoral model in terms of anatomical inspection.

**Methods:**

Six normal porcine femora (180 ± 10 days, 3 lefts and 3 rights) with ball markers were scanned by CT and MRI. The 3D/3D registration was performed by two methods, i.e. the landmark-based 3 points-to-3 points and the surface matching using the iterative closest point (ICP) algorithm. The matching accuracy of the combined model was evaluated with statistical global deviation and locally measure anatomical contour-based deviation. Statistical analysis to assess any significant difference between accuracies of those two methods was performed using univariate repeated measures ANOVA with the Turkey post hoc test.

**Results:**

This study revealed that the local 2D contour-based measurement of matching deviation was 0.5 ± 0.3 mm in the femoral condyle, and in the middle femoral shaft. The global 3D contour matching deviation of the landmark-based matching was 1.1 ± 0.3 mm, but local 2D contour deviation through anatomical inspection was much larger as much as 3.0 ± 1.8 mm.

**Conclusion:**

Even with human-factor derived errors accumulated from segmentation of MRI images, and limited image quality, the matching accuracy of CT-&-MRI combined 3D models was 0.5 ± 0.3 mm in terms of local anatomical inspection.

## 1. Background

X-ray computed tomography (CT) and magnetic resonance imaging (MRI) are based on different physical principles such that they produce vastly different image characteristics [1]. For example, CT can produce the distinct contours of bones, but it cannot show clear images of ligaments. Conversely, MRI shows the shape of both ligaments and bones, but it does not reveal the distinct contour of bones. In addition, an MRI-derived three-dimensional (3D) model inevitably has geometric error originating from its narrow gray scale. Neither imaging modality can produce clear contours of both the bone and the surrounding soft tissues, even though they provide complementary information[2]. To overcome limitations of each modality, a joint model including both hard and soft tissues from CT and MRI imaging information has been demanded, especially for measurement of joint kinematics using model-based 3D/2D registration [1,3,4].

The fusion process of different modalities is typically accomplished by image registration, which transforms the different sets of data into one coordinate system to minimize the differences in specific image features of different modalities. To this end, many 3D/3D registration techniques have been proposed for medical application [4-11]. For the registrations, the use of surface morphology provides more redundancy and is also more cost-effective than using spatial information of landmarks or pixel-wise features of entire volume [12]. Especially, the redundancy of surface morphology may be particularly advantageous for characterizing non-rigid motion [5].

Surface registration can be partitioned into three stages: choice of transformation between two different modalities; elaboration of surface representation and similarity criterion; and matching and global optimization [5]. As regards the surface matching process, the most popular numerical procedure for surface matching is the iterative closest point (ICP) algorithm. The ICP algorithm proposed by Besl and McKay [13] is an iterative descent procedure, which seeks to minimize the sum of the square distance between all points in a source and their closest points in a target model. This algorithm also provides a solution to various free-form surface matching problems, and has been extensively used as an optimizing technique for rigid model based registration in the medical field [10,14-16]. The ICP algorithm requires no extracted features, no curve or surface derivatives, and no preprocessing of 3D data, except for the removal of statistical outliers.

**Figure 1:**
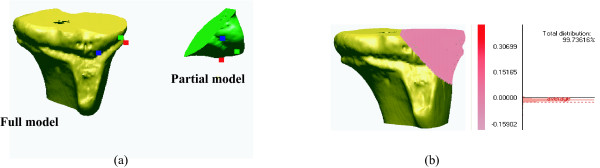
Verification of surface matching with a CT-derived model and its partial model. (a) A full model and a separated partial model, (b) The partial model is registered to its full model

The ICP algorithm is a robust 3D/3D registration method. Our preliminary registration test using the ICP algorithm for this study demonstrated that a part model separated from the full model matched perfectly to its original full model (Fig. 1). In literature[15], the ICP algorithm was able to register a CT-derived bone model to a real patient's bone with an average error of 0.079 ± 0.068° on rotations, and 0.12 ± 0.09 mm on translations; the peak error was 0.288° on rotations and 0.32 mm on translations. Since a registration using the ICP algorithm is robust, it is reasonable to replace the less accurate bone part of MRI-derived models with a more accurate CT-derived bone model using the ICP algorithm.

Thus, we hypothesized that we could build a combined 3D femoral model from the CT- and MRI-derived models with considerably higher accuracy, by using the ICP algorithm. It should be noted that overall small intersurface deviation in a 3D/3D registration does not always coincide with excellent matching in anatomical aspect. Until now, the authors could not find any report commenting on the accuracy of multimodal registration of knee joint model in terms of anatomical inspection. In this study, we quantitatively assess the accuracy of 3D surface matching using the ICP algorithm in terms of anatomic inspection.

## 2. Methods

3D/3D model combination was executed by transferring a CT-derived model to an MRI-derived model and using a numerical surface matching method, i.e. the ICP algorithm. For comparison, landmark-based matching was also executed using a set of three fiducial ball markers on a femoral model. Since the three fiducial ball markers were mounted on the upper side opposite to scanning tables, they were always clearly visible and did not interfere with anything during CT and MRI scannings. Even though it is very difficult to accurately differentiate a bony object in the distal femur from MRI images, there are two areas that we can visually distinguish the boundary between bone and other soft tissues. One is in the femoral condyle (including both medial and lateral condyles) in the distal femur, where the cartilage is thick enough to be distinguished from femoral cancellous bone on MR images. Another area is the middle femoral shaft, where the cortical bone is distinguishable from the surrounding soft tissues, and located in the middle diaphysis not including ephisysis. Therefore, in the current study, the contour-based measurement of matching deviation was performed in those two areas, i.e. the femoral condylar area and the middle femoral shaft.

### 2.1 Reconstruction of CT-derived and MRI-derived 3D models

**Figure 2:**
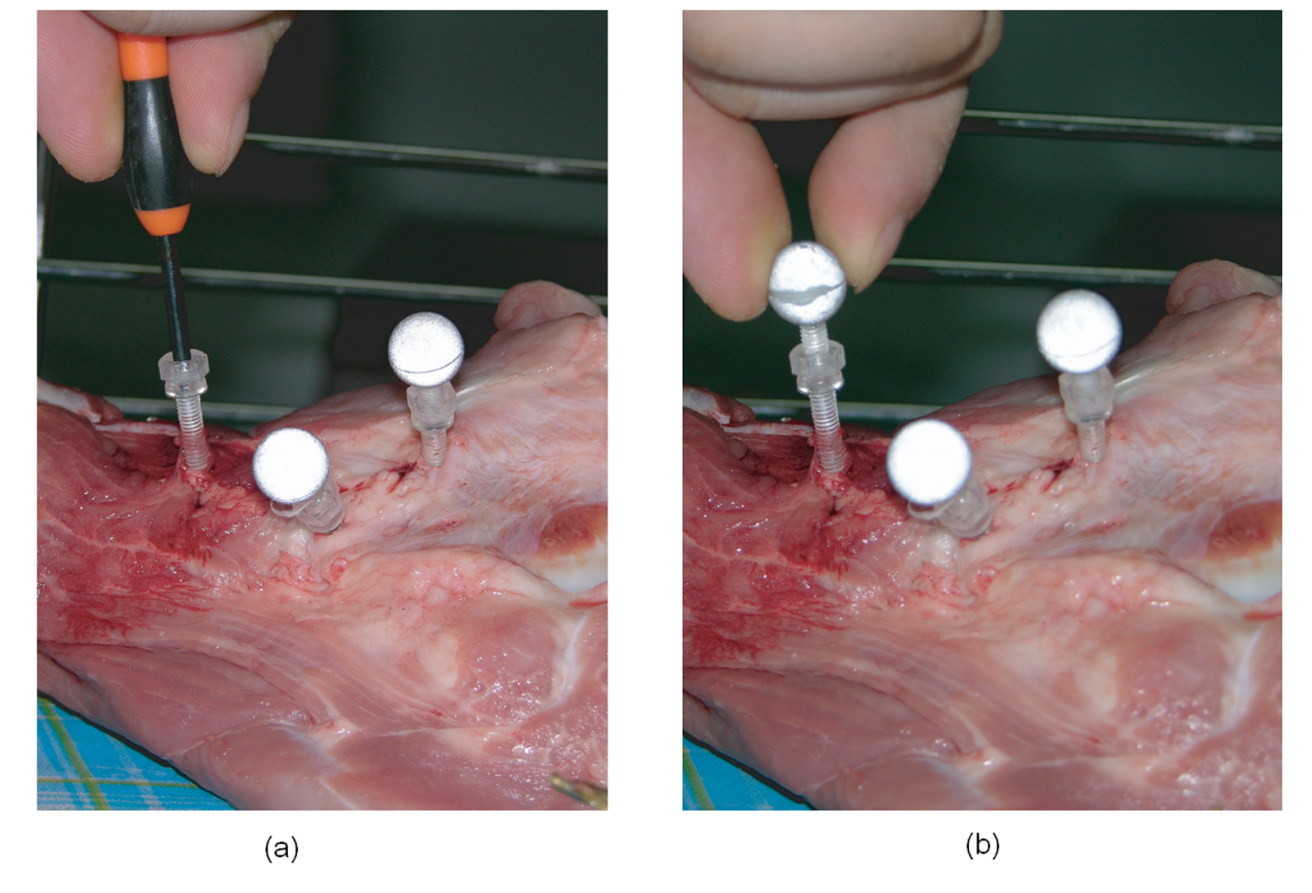
Fixation of Balls. Balls were mounted using 2-stage bolt-nut system which was composed of two plastic bolts of 3.5 mm in diameter and one nut. (a) The 1^st ^bolt was screwed into cortical bone. And a nut was glued on the head of the 1^st ^bolt. (b) The 2^nd ^bolt was connected by the 1^st ^bolt. Finally the balls of 11.5 mm in diameter were mounted on the end of the 2^nd ^bolt by screwing.

Six normal porcine femora from 6 pigs (age 180 ± 10 days, 3 lefts and 3 rights) with ball markers were scanned by a 16-channel CT (GE, USA) and by an MRI Signa Excite 1.5T (GE, USA). Femora were harvested from front legs of fresh frozen pigs. Skin was removed from the femora but subcutaneous soft tissues were kept. CT scanning was performed with 1.25 mm slice thickness and a 0.625 mm reconstruction interval. MRI scanning was done with 1.20 mm slice thickness using fat suppression FIESTA sequence. FOVs of CT and MRI scannings were approximately 230 mm × 230 mm and 200 mm × 200 mm, respectively. The transverse resolutions of CT images and MRI images were approximately 0.3 mm × 0.3 mm (in 768 pixels × 768 pixels) and 0.4 mm × 0.4 mm (in 512 pixels × 512 pixels), respectively. On each femur a set of three optical ball markers (Aesculap, Germany) coated with reflective ink, i.e. ScotchLite 8010 (3M, USA), were firmly mounted with plastic bolts and nuts (Fig. 2). Ball markers were mounted using 2-stage bolt-nut system which was composed of two plastic bolts of 3.5 mm in diameter and one nut. The 1^st ^bolt was screwed into cortical bone. And a nut was glued on the head of the 1^st ^bolt. The 2^nd ^bolt was connected to the 1^st ^bolt by screwing through the nut. Finally a ball of 11.6 mm in diameter was mounted on the end of the 2^nd ^bolt by screwing. Onto each femur, 3 fiducial ball markers were mounted to draw a biggest triangle and obtuse angles. The fiducial ball markers were encapsulated with fat tissue to make the markers distinguishable in MRI images.

CT-derived and MRI-derived 3D models of the ball markers and the femoral models were then reconstructed using Mimics 9.1 (Materialise, Leuven, Belgium). Mimics enables users to paint geometric objects on two other orthogonal anatomical windows as well as on the original scanned window. The CT-derived bone model was constructed based on the image contour with a threshold over 250 Hounsfield Units (HU). Because HU of a bone tissue may slightly differ depending on CT machine, radiation intensity, or artifact due to neighboring tissues, proper range of HU for bone morphology should be calibrated experimentally at each CT scanning event. Therefore, the authors performed HU calibration tests using femoral diaphysis of 10 mm length, and from the test, 250 HU was determined as the proper value for reconstructing an accurate cortical bone geometry from CT images.

The current study designed middle-level skilled segmentation, which can have some small error in contour determination, it should be macroscopically correct. If a non-surgeon having only basic-level knowledge on anatomy has performed contour segmentations alone, the segmented contour may have poor accuracy. Contrary, if an experienced surgeon having deep knowledge on medical images has performed all the segmentations, the segmented contours may be more accurate. Similarly the accuracy of segmentation is dependent on several factors such as anatomical knowledge, segmentation skills, and quality of medical images. In most practical cases, the segmentation is performed by an engineer and a surgeon who have middle-level experience. In the current work, a middle-level segmentation was designed such that the contour of the MRI-based bone model was manually determined by an author (Lee) and macroscopically reviewed by an orthopaedic surgeon with 10-year surgery experience (Seon). Because the errors of this middle-level skilled segmentation may reflect the standard deviation in intra- or inter-observer accuracy, the authors did not test inter- and intra-observer repeatability. After extracting bone contours from the MRI images, MRI-derived 3D bone models were reconstructed based on the slices showing a recognizable bone contour. In case of MRI images, the bone contours were more easily recognized on the boundary between cortical bone and cartilage than in other areas.

### 2.2 Model matching methods

The reconstructed CT- and MRI-derived 3D models were imported into Rapidform 2006 (INUS Technology, Korea). Three ball markers fixed onto a porcine femur were also reconstructed from either CT or MRI images. The center points of the reconstructed ball markers were correspondingly registered as the landmarks onto the CT-derived or the MRI-derived femoral model. The landmarks constructed from CT images were linked to the CT-derived femoral model so that CT-derived landmarks and a CT-derived femoral model could move together with a constant spatial relation. This link between markers and femoral model was also true for MRI-derived models. Before executing surface matching, the MRI-derived model was locked at its original position. Landmark-based measurements and contour-based measurements of matching deviation were executed by using Rapidform 2006 (INUS Technology, Korea).

**Figure 3:**
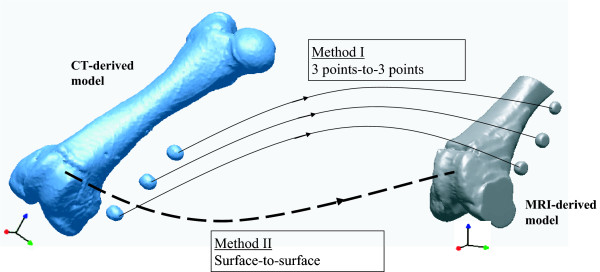
The 3 points-to-3 points matching and the surface-to-surface matching. The 3 points-to-3 points matching was executed by registration the center points of reconstructed CT-derived ball markers to their corresponding center points of reconstructed MRI-derived ball markers. The surface-to-surface matching was executed by 3D/3D surface registration using the ICP algorithm.

Landmark-based matching, generally known as a gold standard matching method for checking accuracy, was executed. The landmarks (center points) of three CT-derived ball models were matched to their corresponding MRI-derived ball models, referred here as the 3 points-to-3 points method (Fig. 3). Because the landmarks and CT-derived model were linked, the reference landmarks reconstructed from CT images were also transferred along the same path as the femoral model. First, the 1^st ^point of the CT-derived model was moved to the its corresponding 1^st ^point of the MRI-derived model. Second, the vector passing the 1^st ^and 2^nd ^points of the CT-derived model was aligned along the vector passing the 1^st ^and 2^nd ^points of the MRI-derived model. Finally, the plane determined by the 1^st^, 2^nd^, and 3^rd ^points of the CT-derived model was placed on the plane determined by the 1^st^, 2^nd^, and 3^rd ^points of the MRI-derived model.

Next, the surface-to-surface matching method implementing the ICP algorithm was performed to match the CT-derived 3D femoral model to the MRI-derived 3D femoral model. Surface matching was completed by using the registration function of Rapidform 2006. In a preliminary test performed in the preparation stage of current study, Rapidform 2006 was judged as a powerful software having excellent 3D/3D registration accuracy (Fig. 1), and it required short computational time. The 3D/3D surface matching by the ICP algorithm was executed in two steps: the initial registration, and the refined registration. In the initial registration step, a user assigned anatomically matched points between the CT-derived and MRI-derived models. The matched points were selected only where bone contours were considered to be accurately segmented from MRI images. The more the user-defined matching points were introduced, the better the initial matching results were [17]. As the second step, the refined registration trial was implemented five times by excluding outliers and applying lower weight to corresponding point pairs of CT- and MRI-derived models. The term outlier was assigned to pairs whose point-to-point distance was higher than calculated standard deviation of the distance, to pairs containing multi-points on end vertices, and to pairs that were not consistent with their neighboring pairs [18]. In the optimization process after excluding the outliers, lower weight was assigned to corresponding pairs with greater point-to-point distance or pairs with large angular differences in their normal directions. In each refined registration trial, pairs of outliers and weighting values were updated. Even though starting positions of objects affected the initial matching accuracy, it did not affect final registration accuracy, since outliers were exclude at the second step.

### 2.3 Accuracy of model matching

The accuracy of the combined model was evaluated by not only landmark-based measurements but also contour-based measurements of geometrical difference between the MRI-derived model and the transferred CT-derived model.

#### 2.3.1 Landmark-based measurements of matching deviation

The landmark-based measurement of matching deviation was completed using landmarks registered at the geometric centers of reconstructed ball markers. The measured matching deviations were spatial discrepancies between the geometric features of CT-derived landmarks and MRI-derived landmarks. That is, the distance between the corresponding landmarks, the interline angle between two corresponding lines connecting two landmarks, and the interplane angle between two planes were all quantified with coordinate values of the landmarks registered to each CT- and MRI-derived model.

### 2.3.2 Contour-based measurements of matching deviation

To confirm the anatomical accuracy of the 3D model matching, the deviation between the MRI-derived model and transferred CT-derived model was evaluated globally and locally. The deviation were in terms of the root mean square distance of the CT-derived contour to the MRI-derived contour. 3D contour-based measure of matching deviation at a polygon node was defined as the shortest distance from a node of the MRI-derived polygon model to the surface of the CT-derived model. And, local 2D contour-based measure of matching deviation was defined as the averaged distance between the 2D sectional bone contours of the CT-derived model and MRI-derived model in a local area. Two local areas were analyzed for the 2D contour-based measurement of matching deviation, i.e. in the femoral condyle and the middle femoral shaft. The femoral condylar area on the capital MRI image showed thick cartilage at the distal-most femur and displayed a relatively clear boundary over the cancellous bone of femoral condyles. The second area was in the middle femoral shaft, in which cortical bone is seen as black in the MRI image taken under the 3D FIESTA sequence, and is clearly distinguished from the surrounding soft tissues.

**Figure 4:**
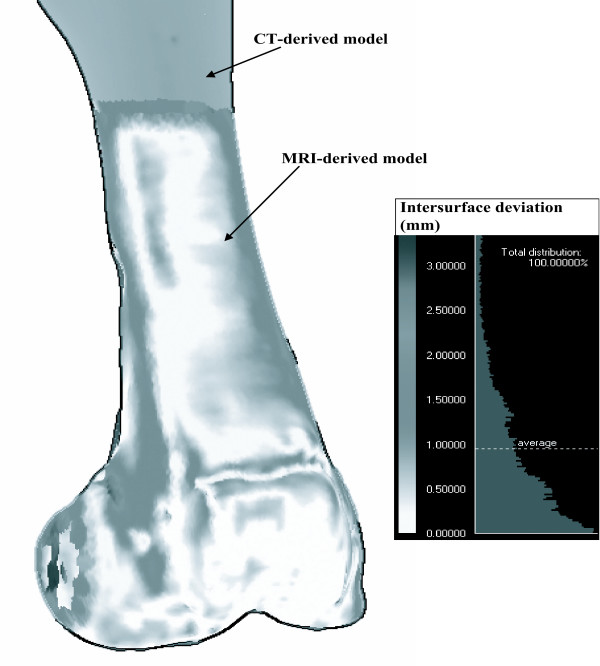
Global 3D contour-based measurement of matching deviation. The contour-base measurement of matching deviation was quantified as the global average of the all distance between the surface nodes of the CT-derived model and MRI-derived model.

Global 3D contour-based measurement of matching deviation was executed by calculating the global average of distances between the corresponding surface nodes of the CT-derived model and MRI-derived model. For visualization, the global 3D contour measure of matching deviation was mapped on the surface of the MRI-derived model (Fig. 4).

**Figure 5:**
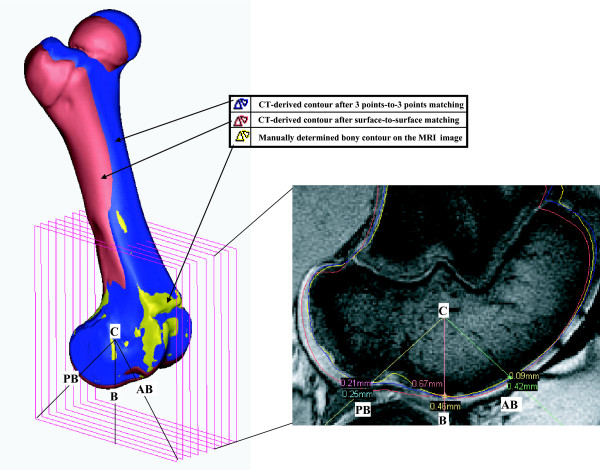
Local 2D contour-based measurement of matching deviation in the femoral condyle. The final value of local 2D contour-based measure of matching deviation was obtained as the average of all the deviation measured at all the intersection pixels on all the sagittal images.

Local 2D contour-based measurement of matching deviation in the femoral condyle was executed on the sagittal MRI images (Fig. 5). On a sectional image at every 2.4 mm along the same medial-to-lateral direction as the CT or MRI scanners followed, 2D contour-based measurement of matching deviation was locally performed. First, in the sagittal view of the femoral condyle, a center line vector was determined. The center line vector was defined as the vector passing the centers of reference spheres that were numerically determined by picking points on the articulation surfaces of the medial and lateral condyles. Subsequently, the anteroinferior vector (the C-AB line), the inferior vector (the C-B line), and the posteroinferior vector (the C-PB line) were drawn in; anteroinferiorly 45°, distally 90°, and posteroinferiorly 45° in the sagittal view, respectively. Each MRI sagittal image was divided by those three vectors. At the points that the matched CT-derived contours intersect with the MRI-derived contour, the local 2D contour deviations were measured. In this way, the final value of local 2D contour-based measure of matching deviation was obtained as the average of all the deviation measured at all the intersection pixels on all the sagittal images.

**Figure 6:**
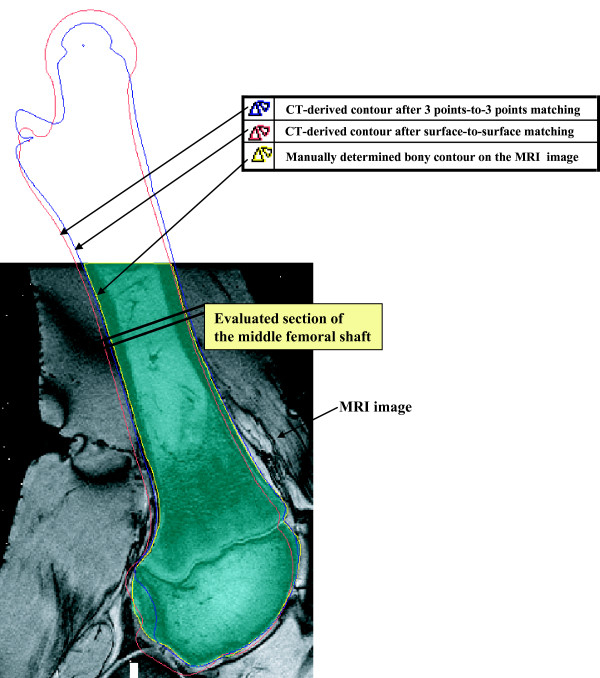
Local 2D contour-based measurement of matching deviation in the middle femoral shaft. The matching evaluation in the middle femoral shaft was done by comparing the inward/outward deviation between the contours drawn on the 2D MRI images.

Local 2D contour-based measurement of matching deviation in the middle femoral shaft was also performed, as the cortical boundary can be recognized on the MRI slices that show a thick bony area. To compare the contour of the CT-derived model with that of the MRI-derived model, the CT-derived model matched was imported to the MRI-derived model with its new spatial information in Mimics 9 (Materialise, Belgium). The contours of the CT-derived models matched were then compared with those of the MRI-derived model. As presented in Fig. 6, the matching evaluation in the middle femoral shaft was done by comparing the inward/outward deviation between the MRI-derived and CT-derived contours drawn on the 2D MRI images. The outward contour deviation of the CT-derived model from the MRI-derived model was measured with a positive sign, whereas the inward contour deviation of the CT-derived model from the MRI-derived model was measured with a negative sign. The sum of the inward and outward deviations was considered as the measure of coincidence between the femoral shafts of the CT-derived and the MRI-derived models.

### 2.4 Statistical Analysis

Statistical difference between the surface-to-surface method and the 3 points-to-3 points method was analyzed in terms of investigated matching deviations. Statistical analysis was performed using univariate repeated measures ANOVA with the Turkey post hoc test. The level of significance (p) was set to 0.05.

## 3. Results

### 3.1 Landmark-based measurements of matching deviation

The landmark-based measurements of matching deviation were performed for both the surface-to-surface method and the 3 points-to-3 points method. The maximum distance error between the corresponding landmarks of the CT- and MRI-derived models was at marker 3, which was averaged to 1.9 ± 1.0 mm (Mean ± SD) for the surface-to-surface method, and 0.6 ± 0.3 mm for the 3 points-to-3 points method. The angular deviation between the corresponding lines of the CT- and MRI-derived models was 1.3 ± 0.8° for the surface-to-surface method, and 0.6 ± 0.6° for the 3 points-to-3 points method. The interplane angular deviation between the reference plane composed of the three landmarks registered to the CT-derived model and that of MRI-derived model was 1.1 ± 0.8° for the surface-to-surface method, and 0.0 ± 0.0° for the 3 points-to-3 points method (Table 1).

**Table 1:** Landmark-based measurements of matching deviation

		Matching	
		
Error item		3 points-to-3 points	surface-to-surface
Distance (mm)	C1-M1	0.0 ± 0.0	1.4 ± 0.8
	C2-M2	0.3 ± 0.2	1.2 ± 0.6
	C3-M3	0.6 ± 0.3	1.9 ± 1.0

Angle (°)	Maximum interline angle	0.6 ± 0.6	1.3 ± 0.8
	Maximum interplane angle	0.0 ± 0.0	1.1 ± 0.8

Note) M1, M2, M3 and C1, C2, C3 are the centers of the reference ball landmarks registered to MRI-derived and CT-derived models, respectively. Distance between two corresponding landmarks registered to CT- and MRI-derived models averaged with standard deviation. Maximum interline angle is the largest value among the angular deviations between corresponding lines connecting corresponding two landmarks. The interplane angle is the angular deviation between the reference plane composed of three landmarks registered to the CT-derived model and that of the MRI-derived model.

### 3.2 Contour-based measurements of matching deviation

In the global 3D contour-based measurements of matching deviation, surface matching using the ICP algorithm (the surface-to-surface method) showed significantly less matching deviation than the 3 points-to-3 points method (p < 0.05). The global 3D contour-based measure of matching deviation averaged out to 1.1 ± 0.3 mm for the 3 points-to-3 points method, whereas 0.7 ± 0.1 mm was the average for the surface-to-surface method (Table 2).

**Table 2:** Contour-based measurements of matching deviation

	Global 3D contour-based measurement of matching deviation (mm)		Local 2D contour-based measurement of matching deviation (mm)			
		
			in the femoral condyle		in the middle femoral shaft	
Specimen	3pt-to-3pt	s-to-s	3pt-to-3pt	s-to-s	3pt-to-3pt	s-to-s
			
1	0.7	0.7	2.2	0.8	0.4	0.4
2	0.9	0.7	2.1	0.5	0.6	0.6
3	0.8	0.7	3.2	0.7	0.8	0.5
4	1.5	0.8	2.9	0.1	1.6	1.1
5	1.4	0.7	6.4	0.5	0.5	0.1
6	1.2	0.8	1.0	0.3	3.0	0.2
			
Average	1.1	0.7	3.0	0.5	1.2	0.5
SD	0.3	0.1	1.8	0.3	1.0	0.3

(Note) 3pt-to-3pt and s-to-s stand for the 3 points-to-3 points matching and the surface-to-surface matching using the ICP algorithm, respectively. SD is the standard deviation.

The local 2D contour-based measure of matching deviation in the femoral condyle appeared to be 0.5 ± 0.3 mm for the surface-to-surface method, which was significantly less than 3.0 ± 1.8 mm (maximum 6.4 mm) for the 3 points-to-3 points method (p < 0.05) (Table 2). Bone contours on the sagittal MRI images near the medial, lateral apexes, and intercondylar notch center were excluded from analysis, since it was not distinguishable from bone due to the lack of cartilage.

The local 2D contour-based measure of matching deviation in the middle femoral shaft was 0.5 ± 0.3 mm for the surface-to-surface method, which was less than 1.2 ± 1.0 mm for the 3 points-to-3 points method (p = 0.18) (Table 2).

## 4. Discussion

This study evaluated the accuracy of the 3D surface matching using the ICP algorithm. Surface matching of the CT-derived models to the MRI-derived models using the ICP algorithm presented a good matching accuracy, an average of 1.1 ± 0.3 mm in terms of global 3D contour matching deviation (Table 2). In addition, local 2D contour matching deviation was as small as 0.5 ± 0.3 mm (Table 2). The combination of CT- and MRI-derived models may have some errors emanating from unclear bone contours of MRI images, observer's lack of knowledge on anatomy. Through middle-level skilled segmentations designed with contour determination of a non-clinician and macroscopic review of an orthopaedic surgeon, current study revealed the CT-derived full bone model can be matched to the MRI-derived partial models with up to 0.8 mm of contour error. Depending on observer, some part of the reconstructed model may have more than 3 mm error. Authors, however, think the registration deviation of 0.8 mm, i.e. a moderate segmentation error, can be achieved for all observers since outliers are excluded during automatic surface matching using the ICP algorithm [19]. And other technical factors can further improve the matching accuracy, if we have more accurate segmentation algorithms of bone contours from MRI images and more reliable 3D matching algorithms.

The 3 points-to-3 points matching using reconstructed ball models is considered not to be a gold standard method for accuracy confirmation. It is because there is accumulated error emanating processes of fixation of markers onto bone through 3D model reconstruction of ball markers. In the case of the 3 points-to-3 points method, the mismatch between the corresponding landmarks registered to the CT-derived model and the MRI-derived model was up to 3 mm (Table 2). In anatomical contour-based measurement of matching, the mismatch of geometric centers of the reconstructed ball markers was significantly larger for the 3 points-to-3 points method than surface-to-surface matching.

The current study evaluated the matching function of the ICP algorithm in building a combined femoral model from CT and MRI images and assessed the accuracy in terms of anatomical inspection. Although alternatives [10,20-22] to the ICP algorithm have been developed since the development of Besl and McKay's [13], the ICP algorithm has remained the most robust and widely used in medical applications [3,21,23-25]. And most of the studies on multi-modal 3D/3D registration techniques [3,10,14,15,21-26] have proposed registration methods without presenting any verification of registration accuracy in terms of actual anatomy. The authors of current study want readers to note that overall small intersurface deviation in a 3D/3D registration does not always coincide with excellent matching in anatomical aspect. In current study, the global 3D contour matching deviation of the 3 point-to-3 point matching was 1.1 ± 0.3 mm, but local contour deviation through anatomically inspection was much larger as much as 3.0 ± 1.8 mm. That is, 3D model fusion with statistically small error can useless when considering anatomic aspects. This is the first report specifically measuring the anatomical accuracy of 3D model matching, when a 3D CT-derived femoral model is matched to a 3D MRI-derived femoral model, in terms of both practical anatomical aspects and the numerical error-distance measurement of matching deviation.

**Figure 7:**
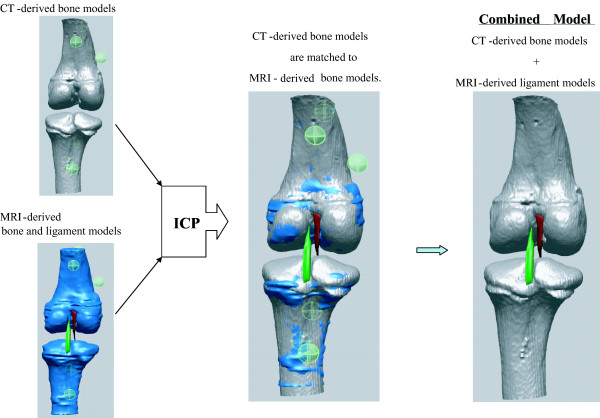
The proposed process of building a combined knee joint model from CT-derived bone and MRI-derived models using the ICP algorithm. Once the CT-derived model is matched to the MRI-derived model, reference geometries of soft tissue attached to the MRI-derived model will be registered to the CT-derived model. Finally a combined femoral model is composed of CT-derived bone models and MRI-derived soft tissue models or reference geometries.

The surface matching of the CT-derived model to the MRI-derived model is considered to provide a good comparison tool in distinguishing anatomical features between CT images and MRI images. With an MRI-derived model containing soft tissues such as ligaments, cartilage, and menisci, the CT-derived bony model will construct a combined joint model taking advantages of both CT scanning and MRI scanning (Fig. 7). Once the CT-derived model is matched to the MRI-derived model, reference geometries of soft tissue attached to the MRI-derived model will be registered to the CT-derived model. With recognition of our measured matching error, one can combine a femoral model that is composed of CT-derived bone models and the MRI-derived soft tissue models or reference geometries. CT-&-MRI bony models includes soft and hard tissues so that they will be used for biomechanical analysis model and volumetric image-navigated orthopaedic surgery system [27].

## 5. Conclusion

This study assessed to what extent the CT-derived femoral model can be accurately matched to the MRI-derived femoral model with help of the ICP algorithm. Even though there might be human-factor derived errors accumulated from segmentation of MRI images, and limited image quality, the CT-derived and MRI-derived 3D models can be combined with 0.5 ± 0.3 mm of contour error in terms of anatomy. And it was also revealed that small statistical global registration error does not mean an anatomically accurate fusion of 3D models. The matching accuracy of the CT-&-MRI 3D model combination implies that CT-&-MRI bony models have the potential to be used as clue materials to build biomechanical joint models including soft and hard tissues.

## Abbreviations

ICP: Iterative closest point; CT: Computed tomography; MRI: Magnetic resonance imaging; 3D: Three-dimensional; 2D: Two-dimensional.

## Competing interests

The author(s) declare that they have no competing interests.

## Authors' contributions

YSL took part in conceiving of the study, carried out experiments and manuscript writing. Jong Keun Seon prepared specimens and carried out experiments. VIS performed 3D/3D registration. GK carried out statistical analysis and data arrangement. MJ reviewed all references and took part in manuscript writing. All authors read and approved the final manuscript.
